# Stable Isotopes for Tracing Cardiac Metabolism in Diseases

**DOI:** 10.3389/fcvm.2021.734364

**Published:** 2021-11-11

**Authors:** Anja Karlstaedt

**Affiliations:** ^1^Department of Cardiology, Smidt Heart Institute, Cedars-Sinai Medical Center, Los Angeles, CA, United States; ^2^Department of Biomedical Sciences, Cedars-Sinai Medical Center, Los Angeles, CA, United States

**Keywords:** metabolism, stable-isotope tracer, metabolic flux analysis, systems biology, cardiovascular disease

## Abstract

Although metabolic remodeling during cardiovascular diseases has been well-recognized for decades, the recent development of analytical platforms and mathematical tools has driven the emergence of assessing cardiac metabolism using tracers. Metabolism is a critical component of cellular functions and adaptation to stress. The pathogenesis of cardiovascular disease involves metabolic adaptation to maintain cardiac contractile function even in advanced disease stages. Stable-isotope tracer measurements are a powerful tool for measuring flux distributions at the whole organism level and assessing metabolic changes at a systems level *in vivo*. The goal of this review is to summarize techniques and concepts for *in vivo* or *ex vivo* stable isotope labeling in cardiovascular research, to highlight mathematical concepts and their limitations, to describe analytical methods at the tissue and single-cell level, and to discuss opportunities to leverage metabolic models to address important mechanistic questions relevant to all patients with cardiovascular disease.

## Introduction

Measuring the dynamic range of cardiac metabolism has been a corner stone of cardiovascular research. Understanding how altered metabolism supports cardiac adaptation during stress and diseases requires a systems-wide approach. Translational models that recapitulate cardiac pathophysiology are critical to advance our understanding of cardiovascular diseases (CVDs) and to utilize metabolic vulnerabilities as biomarkers or for the development of therapeutics. Targeted or untargeted measurements of metabolites allow to assess the metabolic state in an organism *in vivo*. Measurements of metabolic changes in a variety of biological samples are feasible due to advances in chromatographic separation (e.g., hydrophilic interaction chromatography, HILIC; poroshell columns) and improved detection like nuclear magnetic resonance (NMR) and mass spectrometry (MS). However, the interpretation of complex disease models or patient derived samples can be challenging with large scale metabolomics and when a systems-wide understanding of specific pathways is required. Measuring changes in metabolite concentration do not allow to draw any conclusions on metabolic rates or the direction of a flux. Changes in metabolite levels can either result from differential production or utilization of a given intermediate due to increased flux from synthesizing reactions, decreased flux toward consuming reactions, or alterations in transporter activities. For example, during ischemia-reperfusion injury, glycolytic intermediates accumulate despite a reduction in glucose uptake (1–3). Therefore, accurate determination of metabolic flux is necessary for understanding cellular physiology and the pathophysiology of diseases. Steady states in cellular systems are defined by constant values of flux and metabolite concentrations (4). In experimental settings, steady states can be achieved in controlled cell cultures or *ex vivo* perfusions. However, commonly experiments are performed at pseudo-steady state, where changes in flux or metabolite concentrations are minimal over the observed time frame.

**Figure 1:**
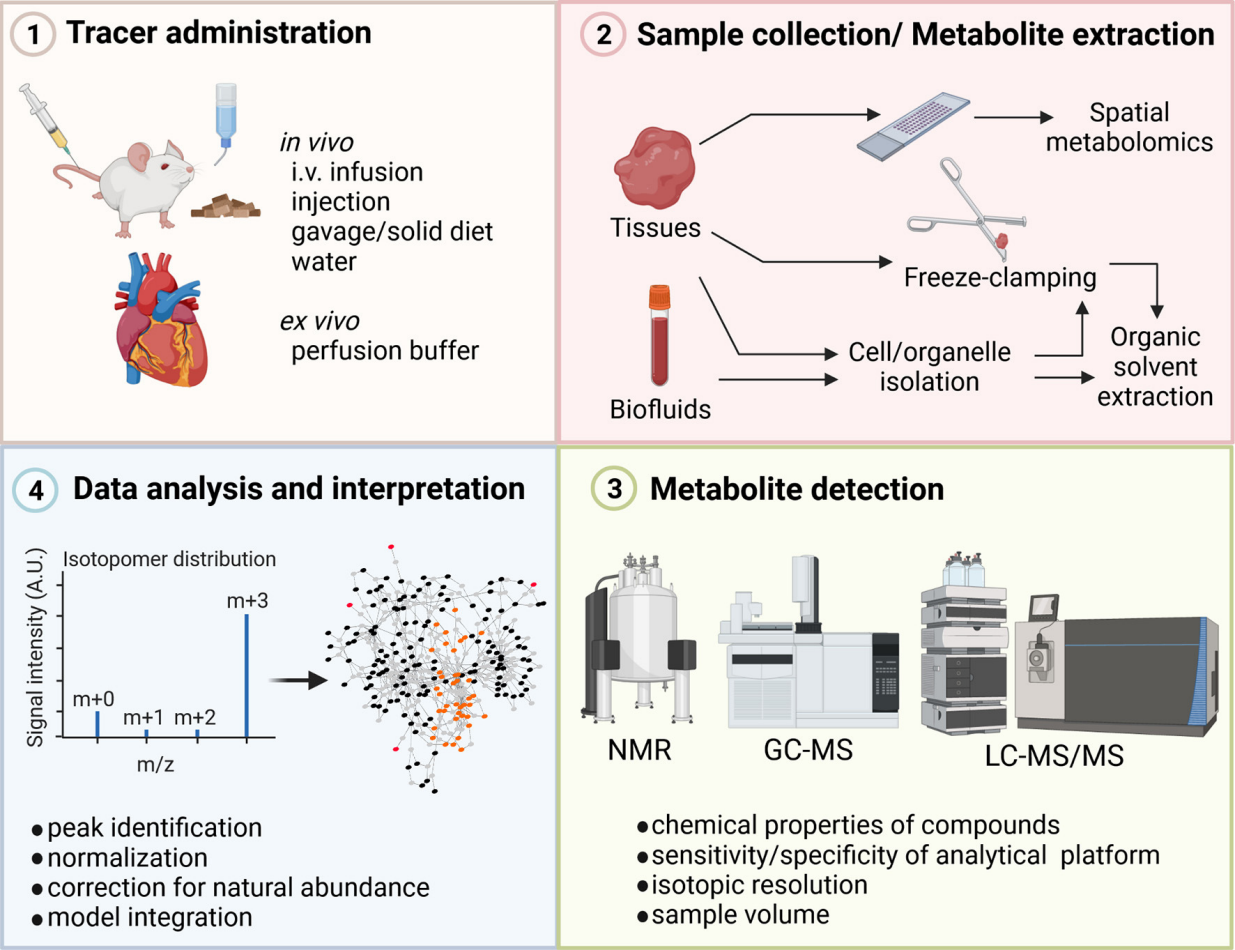
Steps in stable-isotope metabolomics analysis. Stable-isotope tracers to study cardiac metabolism are administered to the model organism or patient using different delivery approaches including, infusion, injections, diet, or *ex vivo* perfusion. Heart tissue or biofluids are collected and metabolites are extracted based on downstream analytical methods. For examples, tissue sample for total metabolite extraction are freeze-clamped in liquid nitrogen and tissue is quenched during extraction using organic solvents. To assess spatial metabolite abundances, tissue slides need to be prepared. Incorporation of isotopic label into metabolites is determined using analytical techniques such as NMR or MS. The isotopic enrichment profile of different metabolites is assessed after normalization and correction for natural abundances. GC, gas chromatography; i.v., intravenous; LC, liquid chromatography, MS, mass spectrometry; NMR, Nuclear magnetic resonance. Figure was created with BioRender.com.

What is metabolic flux? Generally, flux describes the movement of particles across a given area in a specified time. It is important to distinguish between reaction rates and metabolic fluxes. A reaction rate describes the velocity of a given biochemical reaction in response to a substrate and enzyme in isolation, while metabolic fluxes describe the same reaction in the context of a biological system and pathway. Measuring metabolic flux is challenging and cannot be done directly, thus metabolic fluxes are estimations from measurable quantities. Tracer-based approaches provide an apparent straight-forward way of quantitatively assessing dynamic changes in cardiac metabolism. Especially, stable-isotope tracers allow to administer probes to a biological system (e.g., animal cells) in cost-efficient and safe way. At the same time metabolic conversions of labeled nutrients or small molecules allow to track the incorporation of isotopic label (e.g., carbon, nitrogen, or hydrogen) into downstream products and pathways. The detection of specific metabolic products then allows to assess total metabolite changes alongside enzyme activities, flux rates, and overall contribution of specific pathways to the metabolic profile. Tracer studies in isolated perfused murine hearts are commonly used to evaluate metabolic changes in model systems that resemble *in vivo* conditions as closely as possible (5). *In vitro* models using cultured cardiomyocytes have also shown to provide valuable information albeit with a limited scope. The selection of a specific method largely depends on the biological question and inherent limitations of models. Radioactive probes are a stable of both clinical cardiology and basic cardiovascular research (e.g., ^18^F, ^3^H, ^14^C) and are used to study *in vivo* or *ex vivo* metabolic changes in the heart. There are several clinically relevant radiopharmaceuticals such as 99mtechnetium-sestamibi (^99m^TC-MIBI, or CardioLite) (6) or [^18^F]-Fluorodeoxyglucose ([^18^F]-FDG) (7) for interrogating metabolic parameters such as nutrient uptake rates. These probes allow safe, non-invasive tracing of metabolic changes *in vivo*. However, readouts of these measurements are serving as surrogates for multiple metabolic pathways. The desire to measure more in-depth metabolic changes in the heart have led to increased application of stable-isotope tracers in clinical studies (8–10). Stable isotope probes (e.g., ^2^H, ^13^C, ^15^N) are less sensitive than radioactive probes, but in a single experiments stable isotope labeling approaches allow to assess multiple pathways simultaneously over longer experimental time periods. Like radiopharmaceuticals, stable-isotope tracers allow flux assessments, but at the same time these probes provide more in-depth analysis of metabolism both using targeted and untargeted discovery oriented analytical approaches. Increased availability and advancements in analytical techniques for metabolomics have made stable isotope tracers increasingly popular in studying cardiac metabolism *in vivo* and *ex vivo*. In this review, we describe recent advances in applying stable isotope tracers in cardiovascular research and discuss important aspects to consider during data analysis allowing accurate assessments of metabolic changes (Figure 1).

## Stable-Isotope Tracer Methods for Measuring Cardiac Metabolism *in vivo and ex vivo*

Multiple techniques are available for the *in vivo* or *ex vivo* delivery of stable-isotope tracers including oral administration *via* diets, gavage or drinking water, direct intravenous infusion, injection, or perfusion of the heart (Figure 1). Each experimental method has its limitation thus the choice for the specific delivery needs to be driven by the biological question. In *ex vivo* heart perfusions, tracers are delivered via the perfusion buffer. The perfusion technique itself determines how close to physiologic conditions flux rates and metabolic changes can be measured. Two *ex vivo* perfusion techniques for studying cardiac metabolism and muscle physiology are the method of choice: (1) the Langendorff method and (2) the working heart preparation. The aortic perfusion of Langendorff remains a standard preparation and is popular to this date due to its simpler technical requirements and perfusion apparatus (11–14). However, hearts in this preparation do little or no external work and may require external stimulation which can obfuscate metabolic measurements. The more advanced working heart preparation allows arterial perfusion of the heart. This preparation maintains the physiologic contractile function of the heart and prevents periods of anoxia that may occur during Langendorff preparations (15). Several recent studies applied perfusion techniques in a diverse range of disease models to assess cardiac metabolism (Table 1). Regardless of the perfusion technique, tracers are applied through the chemically defined perfusion buffer and allow collection of samples in small time intervals which facilitates dynamic measurements of cardiac metabolism (Table 1).

**Table 1:** Overview of recent *in vivo* and *ex vivo* stable-isotope tracer studies in cardiovascular research, including administration methods, analytical platform, and chromatographic modes.

**Disease/model**	**Organism**	**System**	**Tracer**	**Administration method**	**Analysis**	**Chromatographic Mode**	**Mathematical Model**	**Measurement**	**References**
**Glucose**									
Type II Diabetes	Mouse	Cardiac progenitor cells	[U-^13^C] glucose	Replacement of glucose in cell culture medium	FT-ICR MS	N/A		Incorporation of ^13^C into different metabolites	(16)
Assessing pentose phosphate pathway flux	Mouse	Heart, Liver	[2,3-^13^C]-glucose	Adminstration during *ex vivo* Langendorff perfusion	NMR	N/A		Incorporation of carbons into glutamine intermediates	(17, 18)
Hexosamine biosynthesis pathway	Mouse	Heart	[U-^13^C]-glucosamine	Administration during *ex vivo* working heart and Langendorff perfusion	LC-MS	HILIC		Incorporation of carbons into different metabolites	(19)
			[U-^13^C] glucose						
Mitochondrial pyruvate carrier	Mouse	Heart	[U-^13^C]-glucose	Administration during *ex vivo* Lagendorff perfusion	LC-MS	HILIC		Incorporation of carbons into different metabolites	(20)
					GC-MS	GC			
**Fatty acids**									
Type II Diabetes	Mouse	Heart	[U-^13^C] glucose	Administration during *ex vivo* Lagendorff perfusion	LC-MS	HILIC		Incorporation of 13C into different metabolites	(21)
			[U-^13^C] palmitate						
Absorption of dietary lipids during infancy and adulthood	Mouse	Multiple internal organs	[U-^13^C]-trolein	Intragastric administration of lipid bolus	GC-MS	GC		Incorporation of carbons and hydrogen into fatty acids	(22)
			[U-^2^H]-oleate						
			[1,2,3,4-^13^C]-stearate						
			[U-^13^C]-palmitate						
Doxycyline mediated cardiac dysfunction	Rat	H9C2	[U-^13^C] glucose	Replacement of glucose in cell culture medium	LC-MS	HILIC		Incorporation of carbons into different metabolites	(23)
Perinatal myocardial glucose metabolism	Sheep	Heart	[U-^13^C] glucose	Infusion through fetal tibial artery/inferior vena cava and fetal brachial artery/coronary sinus	NMR	N/A		Determination of AV-differences in the incorporation of carbons into different metabolites	(24)
Nutrient utilization	Rat	neontal cardiomyocytes	[U-^13^C] glucose	Replacement of glucose in cell culture medium	FT-ICR MS	N/A		Incorporation of carbons into different metabolites	(25)
**Glucose/fatty acids**									
Primary carnitine deficiency	Human	whole body assessment	[U-^13^C]-palmitate	continuous intra venous infusion into cubital vein	GC-MS	GC		Measurement of ^13^CO_2_ to determine total fatty acid and palmitate oxidation rates	(26)
			[2-^2^H]-glucose	Bolus intra venous infusion into cubital vein					
Influence of dietary fats	Human	Plasma/breath	[2-^2^H]-palmitate	Continuous intra venous infusion into antecubital vein	GC-MS	GC		Incorporation of hydrogen into NEFA, TAG and lipoprotein-TAG fractions	(27)
		Plasma	^2^H_2_O	Drinking water				Incorporation of hydrogen into VLDL-TAG palmitate	
		Plasma/breath	[U-^13^C]palmitate					Measurement of ^13^CO2 to determine palmitate oxidation rates; Incorporation of carbons into NEFA, TAG, and lipoprotein-TAG fraction	
Propionate-mediated pertubance of cardiac metabolism	Rat	Heart, Liver	[U-^13^C] glucose	Administration during *ex vivo* Langendorff and liver perfusion	GC-MS	GC		Incorporation of carbons into different metabolites	(28)
			[1-^13^C]-palmitate						
			[1-^13^C]-octanoate						
			[U-^13^C]-propionate						
Hexokinase II function	Mouse	Heart	[U-^13^C] glucose	Administration during *ex vivo* Lagendorff perfusion	GC-MS	GC		Incorporation of carbons into lactate, pyruvate, and Krebs cycle intermediates	(2)
			[U-^13^C] palmitate						
**Amino acids**									
Insulin-resistance	Mouse	Multiple internal organs	[U-^13^C]-BCAA	^13^C-BCAA infusion at ~20% of rate of appearance	LC-MS	Amide Column	Modeling of tissue and organ oxidation flux	Incorporation of carbons into tissues and proteins	(10)
Type II Diabetes	Rat	cardiomyocytes	[U-^13^C]-leucine	Replacement of leucine in cell culture medium	GC-MS	GC		Incorporation of leucine derived carbons into different metabolites	(29)
**Multiple tracer**									
Hypertrophy	Rat	Adult Cardiomyocytes	[U-^13^C]-FA mix	replacement of nutrients in cell culture medium	GC-MS	GC		Enrichment of different metabolites in cardiomyocytes or tissue sample and determination of pathway activities	(3)
	Mouse	Heart	[U-^13^C] glucose	13C6-glucose injection after sham/TAC surgery, replacement of nutrients in cell culture medium	LC-MS	HILIC			
	Rat	Adult Cardiomyocytes	[U-^15^N]-aspartate	replacement of nutrients in cell culture medium	LC-MS	Amide Column			
Ischemia reperfusion injury	Mouse	Heart	[U-^13^C]-aspartate	Administration during *ex vivo* heart perfusion	LC-MS	reversed phase		Enrichment of different metabolites	(30)
			[U-^13^C]-glucose						
			[U-^13^C/^15^N]glutamine						
Oxidative stress	Rat	Neontal cardiomyocytes	[U-^13^C] glucose	Replacement of glucose and glutamine in cell culture medium	GC-MS	GC		Incorporation of ^13^C into different metabolites	(31)
			[U-^13^C]glutamine						
Nutrient utilization	Rat	Heart	[U-^13^C]-glucose	Administration during *ex vivo* Langendorff perfusion	LC-MS	C18 reversed phase	Prediction model of isotopomer distribution and experimental validation	Incorporation of ^13^C into different metabolites	(32)
			[U-^13^C]-TAG mix						
Modeling of perfused working hearts	Mouse	Heart	[U-^13^C]-lacate	Administration during working heart perfusion	GC-MS	GC	^13^C-Metabolic flux analysis	Incorporation of carbons into different metabolites	(33, 34)
			[U-^13^C]-pyruvate						
			[U-^13^C]-glucose						
			[U-^13^C]-oleate						
Absorption of dietary lipids during infancy and adulthood	Mouse	Multiple internal organs	[U-^13^C]-trolein	Intragastric administration of lipid bolus	GC-MS	GC		Incorporation of carbons and hydrogen into fatty acids	(22)
			[U-^2^H]-oleate						
			[1,2,3,4-^13^C]-stearate						
			[U-^13^C]-palmitate						
Myocardial Sodium elevation	Mouse	Heart	[U-^13^C]glucose; [1-H]	Administration during ex vivo Langendorff perfusion	NMR	N/A	Flux balance analysis using CardioNet	Incorporation of carbons and hydrogen into metabolic intermediates	(38, 39)

Infusion techniques are likely the most advanced method for the delivery of tracers. Stable isotope tracers are introduced intravenously into the systemic circulation of an animal (e.g., mouse, rat) or human participant. Blood or tissue samples are collected before and after tracer infusion, and isotopic enrichment is then analyzed. Another important factor during stable-tracer experiments is the dynamics of tracers and the determination of metabolite pool sizes (e.g., extracellular vs. intracellular), as well as turnover (or half-life) of metabolites (37). For example, the circulatory system or tubing in heart perfusions can be considered as a single pool. Tracers can be administered (1) continuously or (2) in a bolus. Constant infusion of tracer allows to start sample collections once an isotopic equilibrium is reached, while bolus injection results in an initial increase over time followed by an exponential decrease (37). In either case, pool sizes and compartmentalization of metabolites need to be considered for the estimation of intracellular fluxes (37). Recent studies have shown that pool sizes for metabolites need to be treated as parameters and measured as accurately as possible to improve quality of flux estimations from non-stationary fluxes (38). In metabolomics studies, control and monitoring of the nutrient environment is important. For *in vivo*, special attention needs to be paid to feeding, fasting, diet composition and number of animals per cage. For *ex vivo* studies, special care for the composition of perfusion buffers and reagent purities needs to be taken. For both *in vivo* and *ex vivo* studies it is important to assess if any anesthetic agents may obfuscate metabolic measurements through alterations of plasma metabolite concentrations. The duration of labeling depends on the pathway of interest and whether steady-state data needs to be achieved. In *ex vivo* heart perfusions, steady-state labeling can be reached within 10 to 20 min for key metabolic pathways (39), whereas *in vivo* labeling may require longer timeframes depending on the pathways of interest and tracer (10).

Selecting the right tracer depends on the scope of the study and ultimately which metabolic readout is required for a given biological question. The substrate class (e.g., carbohydrate, amino acid, fatty acid), type of atoms (e.g., ^13^C, ^15^N), number of labeled atoms (e.g., uniform vs. single) and position of labeled atoms will determine which products and pathways will incorporate the label. Any of these options increase the complexity of the experimental design and subsequent data analysis. Selecting the right combination of tracers allows delivering multiple probes and interrogation of multiple pathways simultaneously. Parallel tracing experiments are limited to few applications, but recent advances in high resolution mass spectrometry and development of new probes show promise. For a comprehensive overview of stable-isotope tracers and their readouts, readers are referred to Table 2 in (78). In uniformly labeled tracers, all atoms of interest are substituted with a stable isotope, while single or select position labeled tracers only carry isotopes at specific atoms. The advantage of uniform labeling is better coverage of a variety of different pathways while select position labeling allows to target specific reaction.

**Table 2:** Resources for network reconstruction, simulation and visualization of metabolic flux analysis using stable-isotope tracers.

	**Details**	**Resource link**	**References**
**Metabolic networks of cardiac metabolism**			
CardioNet	Genome-scale metabolic network of mammalian/human cardiac metabolism	https://karlstaedtlab.github.io/cardionet/cardionet	(35, 36)
CardioGlyco	Kinetic model of myocardial glycolysis and oxidative phosphorylation	https://karlstaedtlab.github.io/cardionet/resources; https://www.ebi.ac.uk/biomodels/ MODEL1910170001	(39)
iCardio	Metabolic network of cardiac metabolism based on proteomics information from the human protein atlas and existing human metabolic network reconstructions	https://github.com/csbl/iCardio	(40)
Reactome	Comprehensive open-source pathway database that allows visualization, data integration and interpretation across different data types and organisms	https://reactome.org	(41)
Recon3D	Genome-scale network reconstruction of human metabolic functions; network captures information across organ systems	http://bigg.ucsd.edu/models/Recon3D; https://www.vmh.life/	(42)
TSEM	Tissue-Specific Encyclopedia of Metabolism (TSEM) using the metabolic Context-specificity Assessed by Deterministic Reaction Evaluation (mCADRE)	https://hood-price.isbscience.org/research/tsem/	(43)
**Databases**			
Uniprot	Database for protein sequence and functional information	https://www.uniprot.org/	(44)
Human metabolome	Comprehensive resource and coverage of the human metabolome with biofluid or tissue concentration data, annotation of compounds to reference spectra, chemical structure visualization, chemical taxonomy, and interactive pathway maps	https://hmdb.ca/	(45)
Brenda	Enzyme information database including classification, nomenclature, reaction and specificity, structures, and organism-related information	https://www.brenda-enzymes.org/	(46–48)
KEGG	Kyoto Encyclopedia of Genes and Genomes; database resource for understanding biological systems	https://www.genome.jp/kegg/	(49–52)
**Tools for correction of naturally occurring isotopes**			
MIDcor	Tool for the correction of raw MS spectra for naturally occurring isotopes and overlapping peaks; Requires R	https://github.com/seliv55/mid_correct	(53)
IsoCorrectoR	R-base tool comprising several correction functions	http://bioconductor.org/packages/release/bioc/html/ IsoCorrectoR.html	(54)
IsoCor	Open-source tool for the correction of MS data for naturally occurring isotopes	https://isocor.readthedocs.io/en/latest/#; https://github.com/MetaSys-LISBP/IsoCor/	(55, 56)
**Software for metabolic flux analysis**			
13CFLUX2	Simulation of ^13^C-MFA; allows network modeling, isotope labeling states, parameter estimation and statistical analysis; implementation of cumomer and EMU simulation algorithms	https://www.13cflux.net/13cflux2/	(57, 58)
SumoFlux	Tool integrates modeling and machine learning algorithms to estimate flux ratios from measurable ^13^C-data	https://gitlab.ethz.ch/z/sumoflux	(59)
OpenFLUX	MATLAB-base modeling software for ^13^C-MFA; includes EMU simulation algorithm	https://github.com/lakeeeq/OpenFLUX	(60)
Influx_s	Open-source tool for metabolic flux estimation and metabolite concentrations from stationary and instationary labeling (MFA and INST-MFA)	https://metasys.insa-toulouse.fr/software/influx/	(61)
INCA-MFA	Isotopomer Network Compartmental Analysis (INCA) MFA suite is a MATLAB-based package for isotopomer network modeling and metabolic flux analysis; INCA-MFA allows INST-MFA and constrained based analysis of stable-isotope data	https://mfa.vueinnovations.com/	(62, 63)
SpaceM	SpaceM is an open-source method for *in situ* single-cell metabolomics that integrates microscopy with MALDI-imaging mass spectrometry.	https://github.com/alexandrovteam/SpaceM	(64)
COBRA Toolbox	Constraint-based reconstruction analysis (COBRA) allows the reconstruction, modeling, topological analysis, strain and experimental design, network analysis, and network integration of chemoinformatic, metabolomic, proteomic, and thermochemical data; integration with MATLAB, Gurobi or python	https://github.com/opencobra/cobratoolbox/	(65)
MATLAB	Matlab is a commercial programming and numeric computing platform; Optimization Toolbox^TM^ provides functions for optimization problems including solvers for linear programming, mixed-integrer linear programming, and constrained linear least squares	https://www.mathworks.com	(66)
**Solvers**			
Gurobi Optimizer	Commercial optimization solver for linear programming, quadratic programming, and mixed integer quadratic programming optimization problems	https://www.gurobi.com	(67)
IBM-ILOG CPLEX	Commercial optimization studio to solve complex optimization models	https://www.ibm.com/analytics/cplex-optimizer	(68)
**Programming languages**			
R	R is a programming language for statistical computing and graphics	https://www.r-project.org/	(69)
Perl 5	Perl is a family of high-level, general-purpose, interpreted, dynamic programming languages	https://www.perl.org/	(70)
Python	Python is a high-level, general-purpose, interpreted, dynamic programming languages		(71)
**Network visualization and data integration**			
Cytoscape	Open source platform for the visualization of complex networks and multi-omics data analysis	https://cytoscape.org/	(72)
Metaboverse	Interactive desktop tool for visualization and multi-omics data integration across different species; reactome database integration	https://github.com/Metaboverse/	
**Other useful resources for data analysis and sharing of metabolomics data**			
ChemRich	Tool to analyze metabolomics data based on chemical similarity. ChemRich utilizes chemical ontologies and structural similarity to group metabolites	https://chemrich.idsl.me/; https://chemrich.fiehnlab.ucdavis.edu/	(73)
Chemical Translation Service	Tool for single or batch conversion of metabolite; allows annotation between over 200 databases	http://cts.fiehnlab.ucdavis.edu/	(74)
PubChem Identifier Exchange Service	Tool for single or batch conversion of metabolite within the PubChem database	https://pubchem.ncbi.nlm.nih.gov/ idexchange/idexchange.cgi	(75)
Metabolomics Workbench	International open-access curated repository for metabolomics metadata and experimental data across various species and experimental platforms, metabolite standards, metabolite structures, protocols, tutorials, and educational resources	http://www.metabolomicsworkbench.org/	(76)
MetaboLights repository	Open-access curated repository for metabolomics studies, their raw experimental data and associated metadata	http://www.ebi.ac.uk/metabolights	(77)

**Figure 2:**
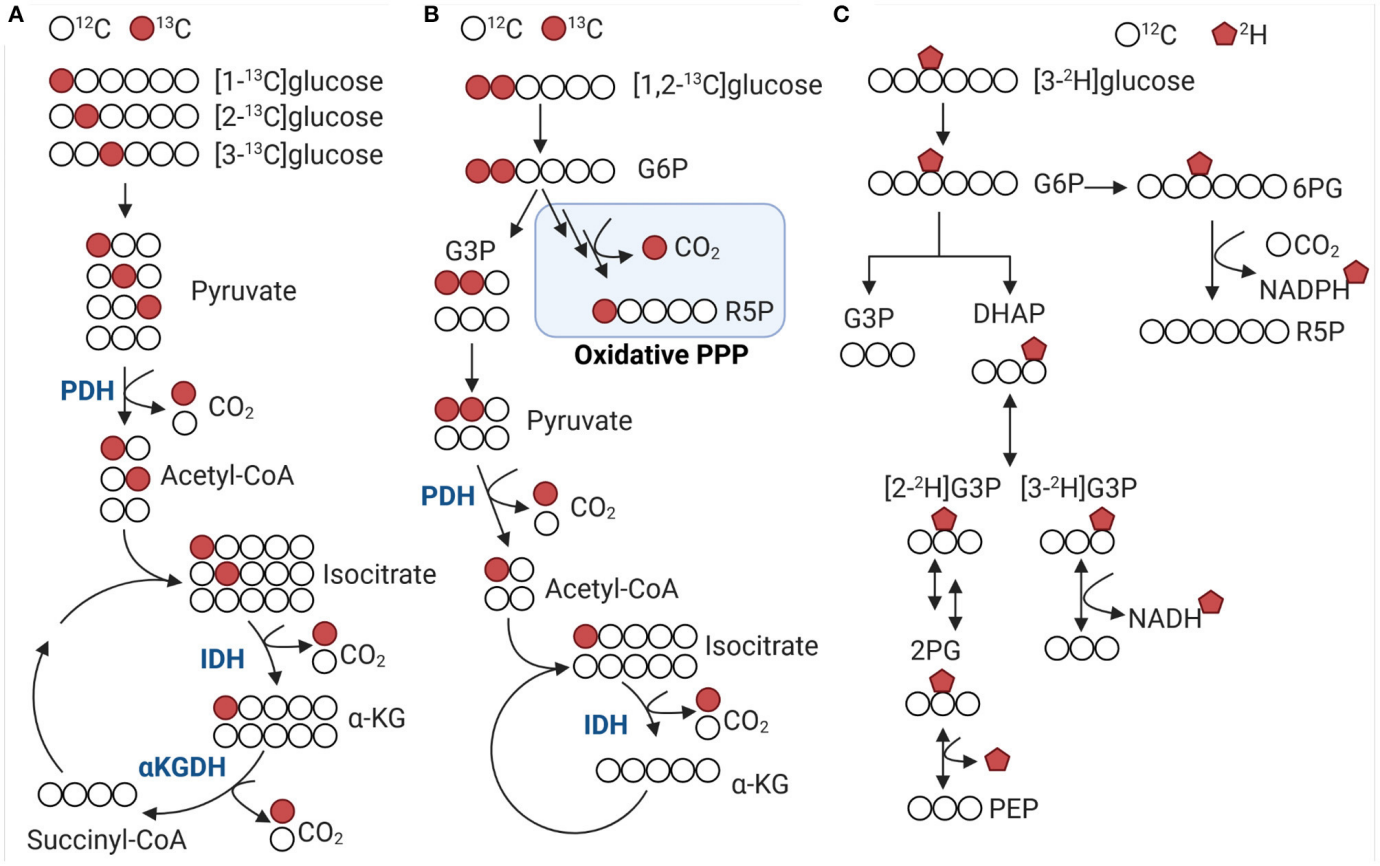
^13^C and ^2^H distribution from labeled glucose in glycolysis and Krebs cycle. **(A,B)** ^13^C-glucose is decarboxylated at different steps in the Krebs cycle and oxidative pentose phosphate pathway. Colored circles indicate ^13^C label for selected metabolic intermediates. [2-^13^C]glucose and [3-^13^C]glucose are associated with pyruvate dehydrogenase (PDH) activity producing m+2 and m+3 acetyl-CoA, respectively. Using [1,2-^13^C]glucose allows to distinguish between glycolysis and oxidative pentose phosphate pathway (PPP) flux. **(C)** [3-^2^H]glucose enters the PPP *via* glucose 6-phosphate (G6P) and decarboxylated to ribulose 5-phosphate (R5P), which generates ^2^H-NADPH. DHAP, dihydroxyacetone phosphate; G6P, glucose 6-phosphate; G3P, glyceraldehyde 3-phosphate; IDH, isocitrate dehydrogenase; α-KG, α-ketoglutarate; α-KGDH, α-Ketoglutarate dehydrogenase; PDH, pyruvate dehydrogenase; PPP, pentose phosphate pathway; 6PG, 6-phosphogluconate; 2PG, 2-Phosphoglycerate; PEP, phosphoenolpyruvate; R5P, ribulose 5-phosphate.

Considering the diversity and versatility of stable isotope tracers, which criteria can we apply to select the optimal probe for a given experiment? The chemical properties, including kinetics, specificity, and turnover, are critical components of tracer selection. Tracer kinetics must be almost identical to unlabeled compounds and probes should not accumulate in tissue. Non-specific binding to other proteins and lipids is generally a concern when using radionucleotides but similar demands need to be met by stable isotope tracers. Lastly, turnover time and tissue concentration of tracers should follow the range of non-labelled metabolites to avoid mass effects and optimal signal-to-noise ratios. Selection of tracers can also be based on metabolic read-outs and targeted toward a pathway of interest. Uniformly labeled tracers of common nutrients (e.g., glucose, fatty acids, or amino acids) can be used to determine how these nutrients are utilized in multiple pathways. Combination with single labeled tracers in parallel experiments then allows to detect specific metabolic fluxes. Different labeling strategies are best described using glucose because of its high abundance, commercial availability, and ease of use in experimental settings. For example, [1-^13^C]glucose can be used to measure the decarboxylation of 6-phosphogluconate to ribulose 5-phosphate in the oxidative branch of the pentose phosphate pathway and the decarboxylation of pyruvate to acetyl-CoA by pyruvate dehydrogenase in the Krebs cycle (Figure 2A). Pentoses produced via the oxidative pentose phosphate pathway can reenter glycolysis via the non-oxidative pentose phosphate pathway branch. Resolution of both glycolysis and pentose phosphate pathway fluxes can be achieved using [1,2-^13^C]glucose tracer (Figure 2B). When glucose is converted through glycolysis, unlabeled (m + 0) and twice labeled (m + 2) pyruvate is produced. Conversion of glucose through glycolysis and the oxidative pentose phosphate pathway produces unlabeled (m + 0), single (m + 1) and twice labeled (m + 2) pyruvate. These flux alterations can occur in the background of increased pentose production via the non-oxidative pentose phosphate pathway which will make it difficult to detect changes in pyruvate labeling (79, 80). Parallel labeling using [1-^13^C], [1,2-^13^C], and [U-^13^C]glucose can improve detection of subtle, aberrant changes in metabolic flux (Figure 2B) (81). Alternatively, [3-^2^H]glucose can also be used to determine redox changes in central carbon metabolism by gaining insight into the *de novo* NADPH synthesis in the oxidative pentose phosphate pathway (Figure 2C) (82). Likewise [2-^13^C] and [3-^13^C]glucose can be used to evaluate the decarboxylation of isocitrate to α-ketoglutarate by isocitrate dehydrogenase and α-ketoglutarate to succinyl-CoA by α-ketoglutarate dehydrogenase (Figure 2A), respectively. Additionally, [2-^13^C] and [3-^13^C] glucose enable determining acetyl-CoA and fatty acid synthesis in the mitochondria and cytosol. The measurement of transporter activities can be achieved by using chemical analogs as tracers. Chemical analogs are compounds that have chemical structures like natural substrates, but with modifications at key positions. These modifications limit how these molecules can be metabolized and lead to accumulation of substrates in tissues. For example, the analog 2-deoxyglucose is transported across the cell membrane and phosphorylated by hexokinase in the same manner as glucose, but because the second carbon has been replaced by a hydrogen it cannot undergo further reactions. As such, 2-deoxyglucose competitively inhibits phosphoglucose isomerase and limits glycolysis. Using analogs 2-deoxyglucose or methyl-D-glucose allows determining uptake flux of glucose. In combination with other tracers it is possible to determine the overall utilization (uptake and metabolization) of glucose. To gain insight into the systems-wide response during disease development, it is also necessary to assess how metabolic alterations are linked to protein dynamics and posttranslation modifications (83, 84). Stable isotope tracers enable measuring posttranslational modifications of proteins. Protein methylation and acetylation can be determined by combining ^13^C-glucose with ^15^N or ^13^C-alpha ketoacids and ^11^C acetate, respectively. The methionine analog, azidohomoalanine, is enables labeling newly synthesized proteins during a short pulse-labeling period (85). The advantage of amino acid analogs compared to ^13^C or ^15^N-labeled amino acids is high sensitivity even at early time points when protein alterations are difficult to identify and often obscured by higher abundant proteins (e.g., structural proteins).

A prerequisite for accurate metabolic measurements is the sample collection and preparation. Metabolites have different turnover rates and differences in their abundance can easily lead to obfuscation of experimental data. For example, rapid degradation of NADPH and CoA derivatives during sample preparation have been well-documented (86, 87). Developing methods and additives to preserve metabolites remains an area of active research. For tissue specimens, freeze clamping using the Wollenberger technique allows rapid freezing of tissue in liquid-nitrogen (88–90). In contrast to flash freezing, this technique has been shown to be superior in homogenously freezing tissue sample and preserving metabolic data. Tissue specimen can then be stored at −80°C for further processing. Similar, metabolite extractions using organic solvents (e.g., methanol:water, 80:20 w/w) quench metabolic profiles and help to reduce enzymatic activities. Measuring more instable metabolites such as NADPH or AMP, a mixture of acetonitrile:methanol:water (40:40:20, w/w) with 0.1 M formic acid can effectively capture and preserve readouts (86, 91). Additionally, adding internal standards during sample extraction can help tracking sample loss and allows correction between batches (87). Independent of the model system or delivery technique, special attention needs to be paid toward standardization of experimental conditions, and sample collection and preparation to ensure rigor and reproducibility of metabolic tracer experiments (76).

## Mathematical Modeling of Metabolic Flux Distributions

Mathematical modeling and computer simulations can help to understand the complex dynamics and interactions of biological processes. A network of biochemical reactions within an organism can be represented by mathematical equations based on established knowledge or extrapolation from other systems. Metabolic models or networks can help to conceptualize and test biological hypothesis *in silico*. In the context of stable-isotope tracer analysis, models are required to estimate flux distributions and explain labeling states for specific metabolites. These modeling approaches can describe the metabolic state of a biological system, but they cannot predict or explain it by identifying regulatory enzymes or principles (e.g., feedback interactions) (4).

**Figure 3:**
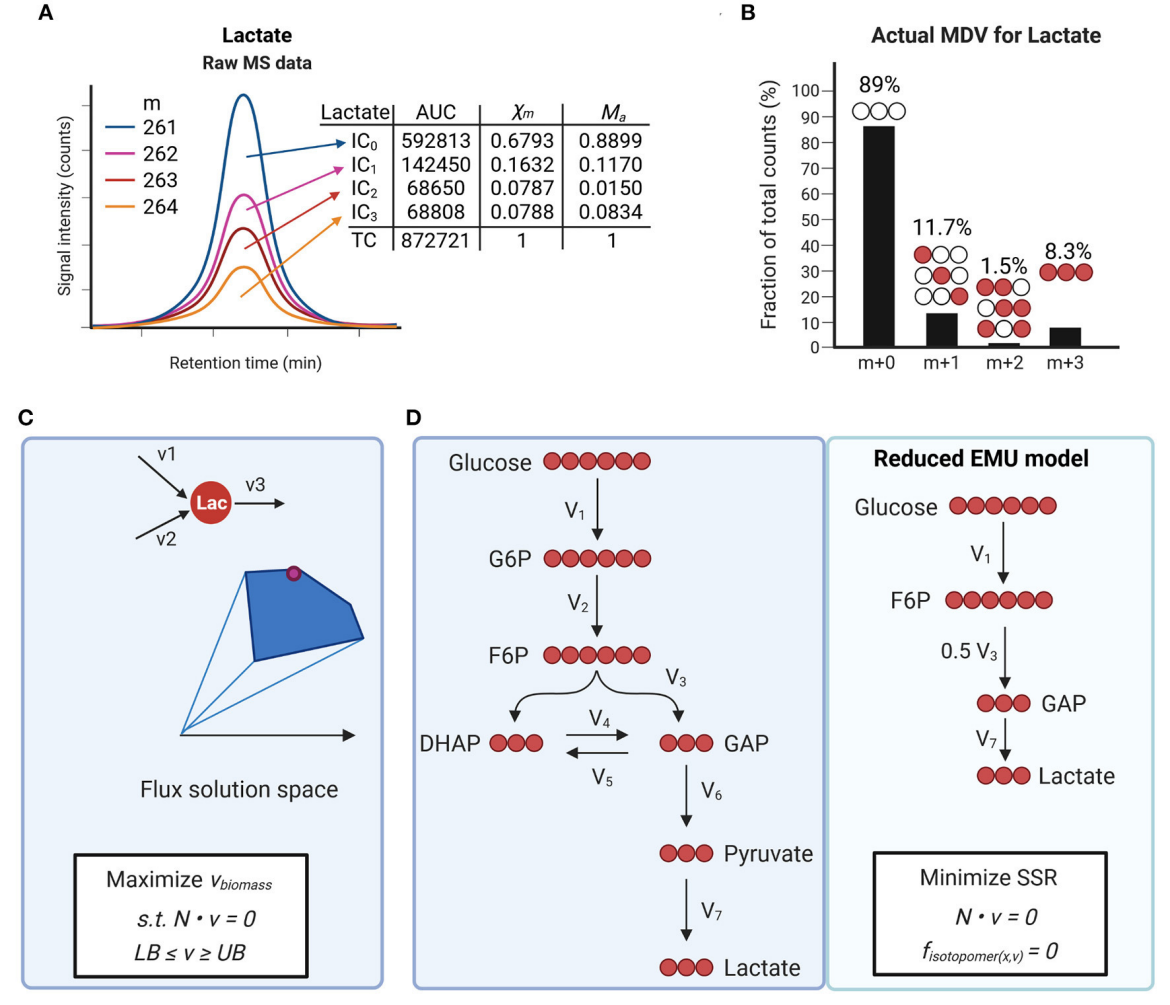
Isotope tracing to measure metabolic fluxes. **(A,B)** Example of isotopomer distribution analysis using mass spectrometry (MS) for ^13^C-lactate. Ion counts for different isotopologues (IC) are determined by measuring the area under the curve (AUC). Mass distribution vectors (MDVs) for lactate (X_m_) are determined by dividing each IC by the total counts (TC). Measured MDVs are then corrected for naturally occurring isotopes **(B)**. Actual MDVs for lactate can now be used for further data analysis such as metabolic flux analysis. **(C)** Flux balance analysis allows estimating flux distributions at steady state. Optimization functions are defined based on biological questions and applied to a network. Constraints for each flux vector are defined as lower bonds (LB) and upper bonds (UB) which allow to define flux solution spaces. **(D)** Schematic of carbon atom distribution in a simplified model of glycolysis using [U-^13^C]-glucose infusion. Network reactions can be further reduced to a define a final reduced EMU model. Flux distributions are estimated by minimizing the sum of least-squared residuals (SSR) between the measured rates and model predicted rates subject to stoichiometric constrain.

Tracer studies can be analyzed using different approaches and level of depth. Most studies determine the relative incorporation of tracer into metabolites and assess qualitative changes in pathway activities. Isotopomers are defined as isomers of a given metabolite that differ only in the mass shift of their individual atoms, for example, ^13^C vs. ^12^C and ^2^H vs. ^1^H in carbon- and hydrogen-labeling studies, respectively. Stable-isotopomer measurements are interpreted by defining mass-distribution vectors (MDVs) that reflect the incorporation of label from a tracer into downstream metabolites (Figure 3A) (57, 92). MDVs are defined as follows:


(1) $$\begin{array}{r} \overset{\to }{X}=\left\{X_{0},\;X_{1},\ldots ,X_{n}\right\} \end{array}$$

(2) $$\begin{array}{r} \overset{n}{\underset{j\;=\;0}{\sum }}X_{j}=1 \end{array}$$

Where each component *X*_*j*_ (with *j* = 0, 1,…, *n*) represents the fraction of a metabolite's pool that corresponds to the *j*th labeled isotopolog and *n* is the maximum number of labeled atoms that a metabolite can incorporate. The fractional isotope enrichments, *X*_*j*_, and their sum equal 1 or 100%. In stable isotope labeling, incorporation of *j* labeled atoms causes a mass shift of *j* atomic units nominal to the mass M of the unlabeled metabolite. Therefore, the MDV is often denoted as follows:


(3) $$\begin{array}{l} \;\vec{X_{m}}=\left\{X_{m0},\;X_{m1},\ldots ,X_{mn}\right\}\\ X_{mj}=\frac{IC_{j}}{TC} \end{array}$$

Where *IC* denotes the isotopolog count for a given metabolite *j* and *TC* denotes the sum of all isotopolog counts. For example, the MDV for ^13^C-labeled glucose can be determined as:


(4) $$\begin{array}{r} {\overset{\to }{X}}_{glucose}=\left\{X_{m0},\;X_{m1},X_{m2,\;}{X_{m3,\;}X_{m4,\;}X_{m5,\;}X}_{m6}\right\} \end{array}$$

with *n* = 6, because a glucose molecule has six carbon atoms where ^13^C-label can be incorporated. *X*_*m*0_ represents the fraction of unlabeled glucose within the total metabolite pool, and *X*_*m*2_ = 0.1 would indicate that 10% of the total glucose pool carries two ^13^C-labeled carbons. Based on the MDV it is not possible to assess where the incorporation of label occurred.

When using stable isotope labeling it is important to correct for natural occurrence of stable isotopes of all atoms within a metabolite. This correction is particularly important when derivatization agents are used during sample preparation (e.g., GC-MS requires derivatization using TBDMS), highly abundant metabolites (e.g., glucose), or single labeled tracers (*n* = 1) are being used. For instance, carbon appears in the form for two stable isotopes, ^12^C and ^13^C, with a natural occurrence of ~98.9 and 1.1%, respectively. When using ^13^C-tracers, measurements need to be corrected for this natural occurrence. Several tools have been developed (see Table 2) (53–56) that allow automatic correction of labeled isotopologues of a given metabolite by solving a linear equation as follows:


(5) $$\begin{array}{r} {\overset{\to }{X}}_{m}={\overset{\to }{M}}_{a}\cdot \overset{=}{L} \end{array}$$

where ${\overset{\to }{X}}_{m}$ and ${\overset{\to }{M}}_{a}$ depict the measured MDV and actual MDVs for a metabolite, respectively. The *j*th row of the matrix $\overset{=}{L}$ contains the theoretically predicted MDV for the *j*th labeled isotopolog. Determining the actual MDVs for each metabolite is a critical step prior to further data analysis and flux interpretation (Figure 3B). To achieve quantitative information about metabolic conversion rates or flux rates different mathematical approaches need to be applied depending on the scope of the study and data type. Techniques in mathematical modeling can be broadly divided into ordinary differential equation and constrained based modeling. For a comprehensive compilation of different modeling techniques and applications, the reader is further referred to Klipp et al. (93).

Flux balance analysis (FBA) is a constrained-based modeling technique that relies on balancing fluxes around metabolites within a network stoichiometry (Figure 3C). Flux distributions in FBA are estimated based on (1) the steady-state assumption, (2) constraints derived from the metabolic environment (e.g., nutrient supply, oxygen consumption), and (3) an objective function such as biomass synthesis, energy provision or another cellular function that is critical for the organ system. Several groups have successfully applied this algorithm and integrated metabolomics data to study cardiac metabolism (35, 36, 40, 94). One limitation of FBA is the requirement for stationary flux patterns without any integration of thermodynamic feasibility. Several methods have been developed to allow inclusion of thermodynamic constraints and even dynamic optimization (95, 96).

^13^C-Metabolic flux analysis (^13^C-MFA) is most widely used in assessing flux in stable-isotope tracer experiments (92). In ^13^C-MFA, isotopomer distributions from labeled substrates (e.g., ^13^C-glucose) are incorporated into a mathematical model of atom transfers and combined with measurements of nutrient uptakes and secretions, and biomass function. The distribution of fluxes throughout a cellular metabolism is mathematically estimated by iteratively solving a least-square regression problem of isotope labeling measurements and extracellular exchanges rates. The models applied to data regression comprise mass balances and isotopomer balances on all network components, thus often encompasses hundreds of equations. The sum of squared residuals (*SSR*) is defined as follows:


(6) $$Minimize\;SSR=\sum \frac{{\left(x\;-\;x_{m}\right)}^{2}}{\sigma_{x}^{2}}+\sum \frac{{\left(r\;-\;r_{m}\right)}^{2}}{\sigma_{r}^{2}}$$

(7) $$\begin{array}{r} N\times v=0 \end{array}$$

(8) $$\begin{array}{r} R\times v=r \end{array}$$

(9) $$\begin{array}{r} f_{isotopomer}\left(v\right)=0 \end{array}$$

Stationary fluxes (*v*) are estimated by the assumption of a steady state (equation 7) and minimizing SSRs between simulated (*x*) and experimentally derived (*r*) MDVs for a given metabolite *S*. The stoichiometry matrix of a given metabolic network is represented by *N*. Uncertainties, σ, can be estimated based on the root-mean square error of unenriched control samples and the standard error of measurement of technical MS replicates. For a metabolite *S*, steady state implies that the total rate at which it is supplied is equal to the total rate at which it is removed (see Figure 3). If the whole system is in a steady state these reaction rates are identical to the corresponding fluxes. Therefore, reaction rates and flux are often used interchangeably (97). In studies using direct infusion or *ex vivo* perfusion techniques, relative fluxes can be converted to absolute fluxes using known infusion rates or flow rates for specific tracers. A prerequisite for applying the above given mathematical concept is that measurements were conducted after reaching isotopic steady state.

A given metabolite comprising *n* atoms may exist in either labeled or unlabeled states with *2*^*n*^ isotopomers. For example, glucose (C_6_H_12_O_6_) consists of 64 carbon atom isotopomers (*n* = 6, 2^6^), 4,096 hydrogen atom isotopomers (*n* = 12, 2^12^), and combined 2.6 × 10^5^ (2^6^ × 2^12^) carbon and hydrogen isotopomers. Therefore, additional methods have been developed to allow the integration of multiple isotopic tracer systems (98). Without these methods, mathematical analysis of tracer studies is often limited to single isotopic tracers to allow efficient simulation of flux distributions. In the elementary metabolite units (EMU) framework labeling information is broken down into multiple individual sub-problems consisting of many coupled non-linear equations (98). The method uses a decomposition algorithm that identifies the minimum amount of information needed to simulate isotopic labeling within a given metabolic network. This approach is different from isotopomer methods, where the model includes all possible isotopomers resulting in an exponentially larger number of variables. The EMU framework requires fewer variables, thus allows the analysis of labeling by multiple isotopic tracers (Figure 3D). For a metabolite comprising *n* atoms, (2^n^ – 1) EMUs are possible. The algorithm allows to identify a minimal set of EMUs to be considered in the simulation model, which reduces the overall number of variables and makes the computational analysis feasible even for multiple-tracer experiments and larger scale models (98). A given metabolic network is decomposed into different blocks of EMUs (98–100), which are expressed as follows:


(10) $$\begin{array}{r} A_{i}\;\cdot \;X_{i}=B_{i}\;\cdot \;Y_{i} \end{array}$$

where matrices *A*_*i*_ and *B*_*i*_ are strictly linear functions of fluxes. In case ^13^C labeling has not reached a steady state, isotopically non-stationary MFA (INST-MFA) methods can be used (62). INST-MFA describes the isotopomer balances using ordinary differential equations (ODEs) rather than linear regression models (92). The advantage of ^13^C-MFA is a higher flux precision and enhanced confidence in the accuracy of estimated fluxes. A typical tracer experiment using [U-^13^C]-glucose can result in 50 to 100 isotopic labeling measurements which are used to estimate just 20 to 30 independent metabolic flux parameters. This leads to redundancy in flux information and ultimately increases confidence.

Several publicly and commercially available software packages have been developed in recent years to facilitate network reconstructions and computational analysis of metabolic network using ^13^C-MFA and INST-MFA methods (62). Table 2 provides an overview of selected resources and repositories, which can be used to analyze stable-isotope tracer studies and may serve as a starting point for readers who are less familiar with including mathematical modeling into their data interpretation. Each of the listed resources serve a different purpose during the metabolomics workflow and in some instances can help with experimental design and selection of tracer probes or analytical platforms (Figure 1). For example, genome-scale reconstructions of cardiac metabolism allow to assess during the study design which metabolic reactions may be involved in the phenotype generation (35, 36, 39). *In silico* simulations can also determine which tracers are required or which metabolites need to be measured for the correct assessment of cardiac metabolic changes. MFA platforms like INCA and SumoFlux allow simulating tracer kinetics, which can be useful to determine when a given tracer may reach steady state under the experimental conditions (59, 63). Here, mathematical modeling can help narrowing done tracer options and provide unbiased analysis prior to experiments. EMU approaches can be used to select tracers for a given network and metabolic pathway by providing one optimal tracer or a reduced list of feasible tracers depending on the probe sensitivity, labeling pattern, and complexity of the metabolic network. Careful consideration of tracer type and labeling pattern prior to an experiment can help reducing potential computational burden of data analysis and improve overall data interpretation by providing clear isotope patterns.

## Platforms for Metabolomics and Single-Cell Analysis

The goal of metabolomics is to comprehensively measure the metabolic composition of a sample in a single analysis. Different analytical platforms combine unbiased, rapid, reproducible, and stable analysis of complex samples in a single run. Each technology has its advantages and disadvantages in terms of sensitivity, throughput, reproducibility, robustness, quantitation, and suitability for specific chemical classes of metabolites. These analytical chemistry techniques range from high-performance liquid chromatography (HPLC), gas chromatography-mass spectrometry (GC-MS), liquid chromatography-mass spectrometry (LC-MS), gas chromatography combustion isotope ratio mass spectrometry (GC-C-IRMS), capillary electrophoresis-mass spectrometry (CE-MS), Fourier transform-ion cyclotron-mass spectrometry (FT-ICR-MS), and nuclear magnetic resonance (NMR). Broadly, analytical chemistry techniques can be divided into (i) separation, (ii) identification, and (iii) quantification of metabolites from a complex sample (e.g., tissue, blood). The separation of metabolites is accomplished using chromatography (GC, HPLC) or CE. Sample detection is then achieved using fluorescence, ion conductivity, or spectrometry (MS, NMR, light absorption). MS and NMR are different types of spectrometers that measure physical characteristics over a given spectrum. MS measures masses within a chemical sample through their mass-to-charge ratio (m/z), while NMR measures the variation of nuclear resonant frequencies. Both detection techniques can be combined with a different separation method allowing customized application methods for a specific type of sample and compound. MS and NMR are widely used in metabolomics to detect and analyze stable isotope tracer studies, such as ^1^H, ^13^C, ^14^N, and ^31^P. A MS instrument consists of three main components: an ion source, a mass analyzer, and a detector. The ion source ionizes the sample, producing ions and fragment ions, then accelerated through the mass analyzer. Perpendicular magnetic fields deflect ions traveling through the mass analyzer according to their mass allowing to sort and detect ions based on their mass-to-charge ratio (m/z). The output is presented as a mass spectrum where each peak represents a different ion with a specific m/z, and the peak length corresponds with a relative abundance. Combining MS with separation techniques like GC or LC enables analysis of complex samples based on separation times (or retention times) and m/z ratios. The variable ionization of compounds, ion suppression, and instrument cycling times inherently limit the number and type of metabolites that can be distinguished in a single run. Different ionization techniques have been developed including electron ionization (EI) and chemical ionization (CI) for compounds in gas-phase, as well as electrospray ionization (ESI) and matrix-assisted laser desorption/ionization (MALDI) which are suitable for thermally labile and non-volatile analytes (101). After ionization, the mass analyzer sorts the ions by their m/z ratio using magnetic or electrical fields. Common analyzers use time of flight (TOF), quadrupole mass filter, quadrupole ion trap, Fourier transform-ion cyclotron or orbitrap. Each separated ion induces a specific charge which is finally measured in the detector. The advantage of MS over NMR is higher throughput, sensitivity, analysis speed, and a broader range of applications. Continuous improvements in instrumentation design and reduced running costs have led to increased accessibility and implementation of MS in research studies. The sensitivity and resolution of NMR instruments depend on the magnetic field strength, which has been improved in the past decade. ^1^H NMR spectra have a small chemical shift range, which leads to overlapping peaks in complex samples, limiting detection, and lowering sensitivity. Combination of one-dimensional with two-dimensional NMR spectrometry has improved signal dispersion and compound identifications (102). Implementation of cryo-or microprobes further reduced scanning times needed to record a spectrum and greatly improved the sensitivity. Another advantage of NMR spectrometry is the quantitation and more uniform detection system, which can be directly used to identify and quantify metabolites both *ex vivo* and *in vivo*. In addition, the non-destructive nature of NMR methods leads to simplified sample preparation or even enables direct measurement of samples from body fluids (e.g., urine). Another advantage over MS is quantifying multiple compounds without the need for calibration curves for each compound. MS and NMR spectrometry have evolved as the most common techniques in stable isotope tracer and metabolomics studies. However, there is no single analytical platform that can achieve a complete quantification and identification of all molecules within a sample. Therefore, more than one method must be employed for comprehensive metabolic profiling. When deciding on an analytical avenue, the choice primarily depends on the focus of the research study and the nature of the samples, and instrument and know-how accessibility.

Single-cell analysis is a powerful tool to interrogate cellular heterogeneity within the same tissue, allowing for refined assessment of phenotypes and biomarker discovery. Metabolomics at the single-cell level holds the promise to obtain precise spatial and temporal information allowing to assess cell differentiation and division, cell-cell interactions, metabolic cooperation between cell populations and a detailed stress response analysis. In comparison to other omics technology, single-cell metabolomics faces several challenges because of the chemical diversity of metabolites, wide range of concentrations, sample stability, and lack of amplification (103). Recent studies have tried to circumvent challenges in single-cell metabolomics by using co-immunoprecipitation of proteins followed by MS to capture metabolic features (104). These strategies raise the question how much of metabolic regulation is dependent on protein abundance changes. The relationship between enzyme function and metabolites is multifactorial and dynamic, which explains poor correlation between individual proteins and metabolites (105, 106). Integration of proteomics and transcriptomics data with mathematical modeling and machine learning algorithms may improve the predictive value of these methods (107). Metabolomics at the single-cell level includes several pre-processing and sample conditioning steps, including desalting, to decomplexify the sample. These additional steps increase robustness but can also lead to sample loss. The limiting factor in stable-isotope labeling for single-cell analysis is sensitivity and isotopic resolution of a given analytical platform. Detection of metabolites in small sample volumes has been achieved with both NMR and MS (108). However, the sample volume for NMR application is larger than the single-cell level, thus NMR is commonly used with tissue samples or body fluids (e.g., plasma, urine). In recent years, progress has been made to facilitate mass spectrometry-based single-cell metabolomics through increased instrument sensitivities and enhanced technologies. Mass spectrometry Imaging (MSI)-based methods are currently the most sensitive, thus preferred analytical platforms for single-cell metabolomics (103). MSI is a logic progression of laser microdissection technology and combines high-resolution microscopy with MS, which can be applied to thin tissue section or dispersed cell population attached to a grid. MALDI-MSI and ESI-MSI are the two conventional platforms applied for single-cell metabolomics. Both techniques allow spatial resolution of very small sample amounts (μl to pL range), analysis in the attomole ranges, and integration with automation. Recent studies demonstrate that these MS technologies can be applied to identify metabolic alterations in endothelial cell migration (109), tumor cell metastasis (110), and even to the single organelle level (111). The complementary nature of single-cell approaches enables spatial characterization of different cell types, reproducible measurement of metabolic states and organelle analysis when combined with other electrophysiological (e.g., patch clamping) techniques. However, single organelle analysis remains challenging due to limitations in reproducibility, low analyte abundances, limited sample volumes, and interference from sample impurities (103). Single-cell metabolomics is feasible across different platforms due to recent advance in MS technology, which enables enough resolution for monoisotopic detection. For studies that do not require a spatial resolution but molecular characterization at the single-cell level, separation-based methods like capillary electrophoresis (CE), LC, or GC combined with MS or fluorescent tagging approaches can provide sufficient sensitivity for certain applications (112). Analytical platforms like CE-ESI-MS allow qualitative and quantitative analyses of single cells and subcellular compartments with high resolving power and low sample input (<1 μl). CE is a powerful platform and can be coupled with optical, electrochemical, or MS-based detection expanding its applications (112). Compared to other application, one limitation of CE is its low throughput (113). Separations using CE can last up to 1 h, which limits the number of cells that can be assayed from one population (114). Therefore, recent methods have been introduced that directly inject cells into the capillary for lysis and separation reducing the time between cell rupture and analysis. Further advances in MSI and separation-based MS for single-cell metabolomics will offer unique approaches to classify cell types and identify subpopulation thus enhance our understanding of metabolic remodeling during cardiovascular diseases.

## Concluding Remarks and Future Challenges

Understanding how reprogrammed metabolism supports cardiac adaptation during stress and diseases requires a systems-wide approach. Translational models that recapitulate cardiac pathophysiology are critical to advance our understanding of CVDs and to utilize metabolic vulnerabilities through the development of novel therapeutics or biomarker identification. Stable-isotope labeling and mathematical modeling require multidisciplinary collaborations to bridging animal models into patients. Likewise, clinical studies should inform animal models for mechanistic hypothesis-driven testing. Challenges persist in accurate estimations of fluxes from stable-isotope tracers and integration into clinical trials. Evidence indicates that metabolic phenotypes are a key determinate of disease development and progression. Advancements in analytical methods to quantify metabolic phenotypes *in vivo* will be critical to identify metabolic vulnerabilities. Ultimately, these efforts may help clinicians to tailor therapeutic interventions based on the metabolic profile of the intact heart.

## Author Contributions

AK designed and wrote the manuscript.

## Funding

This research was supported by R00-HL-141702 and institutional funds from Cedars-Sinai Medical Center to AK.

## Conflict of Interest

The author declares that the research was conducted in the absence of any commercial or financial relationships that could be construed as a potential conflict of interest.

## Publisher's Note

All claims expressed in this article are solely those of the authors and do not necessarily represent those of their affiliated organizations, or those of the publisher, the editors and the reviewers. Any product that may be evaluated in this article, or claim that may be made by its manufacturer, is not guaranteed or endorsed by the publisher.
